# Dropout in youth and adult education: a multilevel analysis of students and schools in Chile

**DOI:** 10.3389/fpsyg.2023.1163088

**Published:** 2023-06-01

**Authors:** Tabata Contreras-Villalobos, Verónica López, Enrique Baleriola, Luis González

**Affiliations:** ^1^Escuela de Psicología, Facultad de Filosofía y Educación, Pontificia Universidad Católica de Valparaíso, Valparaíso, Chile; ^2^Departamento de Psicología Básica, Evolutiva y de la Educación, Universitat Autònoma de Barcelona, Barcelona, Spain; ^3^Centro de Investigación para la Educación Inclusiva, Viña del Mar, Chile; ^4^Estudis de Psicologia i Ciències de l’Educació, Universitat Oberta de Catalunya, Barcelona, Spain

**Keywords:** school dropout, school abandonment, youth and adult education, multilevel analysis, Chile

## Abstract

**Introduction:**

School dropout is an increasing worldwide phenomenon, marked by inequality and educational exclusion. In Chile, many students who have dropped out of regular schools attempt to reenter youth and adult education (YAE). However, some of them drop out again from YAE.

**Objective:**

The aim of this study was to identify and jointly analyze school and individual factors that influence dropout in YAE.

**Methods:**

This secondary multilevel analysis of official datasets from Chile’s Ministry of Education focused on students enrolled in YAE (*N* = 10,130).

**Results:**

According to the findings, YAE dropout can be explained by the individual risk factors of age (19–24 years), low academic achievement, and school-level factors such as number of teachers (raw and student-to-teacher ratio), economic resources, and school management quality.

**Discussion:**

We discuss the need to develop school-level protective factors that build connections, foster student engagement, and ultimately, promote students’ permanence and progress in YAE.

## 1. Introduction

Recent years have seen international **organizations** call for education policies that improve the quality, equity, inclusiveness, and fairness of teaching and school processes (UNESCO, 2015). **However**, many gaps remain to overcome, such as access to education and school completion, regardless of students’ educational pathways (OECD, 2012; de Cristo, 2019). In this regard, more than 12 million children, young people, and adults are excluded from education worldwide (UNESCO, 2020). This issue exacerbates the education crisis, translating into exponential growth in school dropout rates (EduCo, 2021).

School dropout, or leaving school, is broadly defined as people who discontinue their studies (European Commission, 2003). It is used as an indicator to measure the relationship between academic progress, age, and education level. Students who are unable to keep up may leave the education system, contributing to the school dropout rate (Camacho, 2016). School dropout is not only an indicator; it also has negative and far-reaching effects on people’s lives in terms of finances, employment, mental health, wellbeing, cohesion, and social engagement (Bae, 2020; Andrew and Blake, 2021). Thus, school dropout is regarded as the outcome of a process marked by school failure, demotivation, and disengagement with school and the education system (Jabbari and Johnson, 2021). Its precursors are absenteeism, low performance, failure, grade repetition, and educational backwardness (Jurado and Tejada, 2019). Hence, school dropout is a major cause of inequality and social exclusion and one of the most entrenched phenomena in education systems, affecting millions of vulnerable young and adult people (Lee-St et al., 2018; Tarabini, 2020; Contreras-Villalobos et al., 2022).

Many of those who drop out of school graduate later than usual (OECD, 2018). They often complete their studies in another educational setting, namely, youth and adult education (YAE) programs. The institutions offering these programs are essential because they strive to get people—mostly those younger than 25 years of age—back into school so that they can complete their compulsory education. However, despite their good intentions, YAE schools confront another challenge with respect to school abandonment. Chile is a paradigmatic case because although 1.7% of students drop out of regular education, this figure rises to 24% in YAE (MINEDUC, 2020). In other words, almost 34,000 people drop out again each year after having reentered the school system via YAE without receiving further intervention or support to finish their compulsory education.

This means that between 5.2 and 8.9% of the population between the ages of 6 and 21 years old who once entered the education system have dropped out and have not since returned (de Cristo, 2019). The inexactitude of these figures is a consequence of the disengagement and invisibility of YAE students, which are a result of the existing segregation of the most vulnerable groups (Verger, 2019). This scenario has become even more complex in the wake of the COVID-19 pandemic, given official figures estimate that school dropout rates will increase by 200% (MINEDUC, 2020).

School dropout in the context of YAE is even more complex, with higher figures than in mainstream education. So, in this context, two questions arise: What variables are decisive in understanding school dropout in YAE? What actions can be deployed to intervene in this phenomenon? To answer these questions, we carried out a multilevel exploration of the underlying factors to identify lines of action to address this situation (Bronfenbrenner, 1992). Our research objective, therefore, was to identify and collectively analyze individual and school factors that affect school dropout in Chile’s YAE programs. Through this line of study, we generated recommendations on how to address this problem, which affects not only young people but also their social environments.

### 1.1. Managing school dropout in YAE

School dropout requires urgent attention at different levels. Action is needed at the political level, given its direct consequences on young people and adults. These effects include reduced social cohesion, less engagement and civic participation, and threats to their educational trajectories, entry into the labor market, and economic development. These threats exacerbate inequalities and social vulnerability, affecting students’ pursuits in life (Causton-Theoharis et al., 2011; UNESCO, 2017). In this way, school dropout in YAE must be addressed, not only to provide an opportunity to complete compulsory education, but also to promote more comprehensive personal and social development, including non-cognitive or relational aspects (Nada et al., 2020; Paniagua, 2022).

In Chile, YAE falls under the oversight of the Ministry of Education. It was originally created to give adults an officially recognized way to pursue their education. However, due to cultural, social, and economic shifts, it is currently used more by young people, 75% of whom are between 15 and 24 years of age (MINEDUC, 2017), a phenomenon that is seen worldwide (Novella, 2020).

Such institutions are internationally regarded as high-complexity schools, given the high-risk social environments their students face (Murillo and Duk, 2020). However, the students are not the only ones coping with adverse circumstances: The institutions themselves often encounter complex conditions that lead to an exclusionary organizational environment, scant resources, and frequent reliance on self-management (Opazo, 2017). In Chile, YAE institutions’ capacity for action is limited due to the short supply of basic funding and lack of an education policy that might otherwise provide additional grants. These schools do not currently have enough fixed resources to plan and execute effective responses to the needs of their students (MINEDUC, 2016; Rodríguez, 2019). The low-coverage, unsustainable, swing-budget project funding model and attendance-based grant system currently in place further challenge public education (Bellei, 2015).

Youth and adult education policies are currently designed around high-stakes testing, privatization, and demand-side grants that determine the resources schools must have to operate, further deepening inequality in this type of education (Author; Mikulec, 2021). This model affects school management, creating new problems and challenges for both institutions and their students. Hence, strategic planning is needed to comprehensively monitor and assess these institutions to understand and manage the work they do to prevent students from dropping out (Grinberg, 2006; Quiroga, 2017).

At the school level, research on school dropout has shown that school management is key in tackling this problem (Mduma et al., 2019; Contreras-Villalobos et al., 2022). Certain factors at the institutional level can help keep students in school, including policies and legal regulations, resources (Alvariño et al., 2000), and school governance that determine how schools are **organized** and managed (Ball, 2000).

In turn, school-level management determines educational practices and nurtures environments that benefit learning and help keep students on their educational pathway, albeit not always with the same level of success (Bolívar, 2019). Important factors include physical arrangements (Veiga-Neto and Noguera, 2010), the availability of learning means and resources, the size of the school, the number of people it can accommodate, the opportunities it provides for gathering and participation, and even the educational practices, including whether they enable personalized work that emphasizes recognition and representation (Belavi and Murillo, 2020).

However, in recent years, the scientific literature has identified certain factors that are complex to establish or measure yet are key in the school’s management of dropout. Among these factors is the bond between teachers and students as a protective element, whereby pedagogical work, communication, and individualized work generate transformations in educational trajectories (Farmer et al., 2018). This situation is highlighted in alternative schools, where new opportunities for learning are generated, providing support to students at risk of school dropout and shaping new experiences in the school environment (Wilkerson et al., 2016).

At the individual level, indicators of disengagement between students and the institution should be addressed if dropout is to be tackled by school management. Among the most frequently mentioned in the literature are poor attendance, grade repetition, perceived school failure, financial struggles, family burdens, behavioral difficulties, certain geographical contexts, and drug use (Opazo, 2017; Tarabini, 2017; Rodríguez, 2019; Valenzuela et al., 2019).

However, the complex nature of school dropout demands an integrative perspective that analyzes educational management across all levels. It is impossible to consider the impact of any of these dimensions or factors in isolation because how schools are run is linked to macro-level social, cultural, and political contexts and the micro-level circumstances of individual students and their immediate environment (Bronfenbrenner, 1992; Castro, 2008). Hence, a comprehensive, structural approach is needed to analyze school dropout in YAE because it allows moving away from reductionist, instrumental, or mechanistic interpretations to provide insightful recommendations for transforming school and public policies. For this reason, this research explored factors related to school dropout in YAE at the school and individual levels to determine their attributed variability. This allowed us to identify lines of action for the transformation and improvement of school dropout.

## 2. Materials and methods

To accomplish the explanatory objective of this research and address the hierarchical nature of the data, we performed a multilevel analysis, an extension of linear regression models that recognizes and accounts for the nested nature of data. This type of analysis allowed us to ascertain the proportion of variance explained by differences between cases (students) in a certain context (schools)—known as within-school variance—and compare it with the proportion of variance explained by differences among contexts (schools)—known as between-school variance (Martínez-Garrido and Murillo, 2013).

### 2.1. Participants and materials

The analysis was conducted with a national sample of 10,130 students (51% men; age: *M* = 24.2 years, *SD* = 9.6) from 179 schools in 11 regions in Chile. These 179 schools had an average of 56.6 students enrolled (*SD* = 72.5; Min = 3; Max = 592), where 88.3% were public municipal schools, 10.0% were subsidized private schools, and 1.7% were public education local services. Although the Ministry of Education’s (MINEDUC, 2020) records indicate more students enrolled in YAE nationwide (*n* = 139,581), information is not available or complete on many of them. Therefore, the sample only included students for which complete information had been provided by schools to the central government.

### 2.2. Instruments and variables

We consulted official public datasets from the Ministry of Education, available in the open data section of its online study center. Using this platform, we requested four datasets for 2018: (a) performance summary, (b) enrollment summary by school, (c) national performance evaluation system, and (d) school grants. Table 1 identifies the datasets used to derive certain information and how variables were operationalized for the two levels of our analysis: students (level 1) and schools (level 2; Woltman et al., 2012). Based on the information available and previous empirical studies, we hypothesized that school dropout would be explained at the individual level by gender, age, and grade point average (GPA). This implies that young men (≤19 years old) with low academic performance would have the highest risk of dropping out a second time during YAE. At the school level, we expected that depersonalized work, low school resources, and hierarchical and standardized school management would make the highest contribution to explaining school dropout in YAE.

**TABLE 1:** Operationalization of variables.

Level	Variable	Operationalization	Data source	Type of measure	Response options
Individual	School dropout (abandonment)	Understood as a student’s status at the end of the 2018 school year, i.e., whether they completed the year or withdrew and did not enroll at another school during the same year	Performance summary, 2018	Nominal	Passed; failed; dropped out
	GPA	Student’s overall grade point average at the end of the school year	Performance summary, 2018	Continuous	1–7
	Gender	Identifies a person as man or woman according to patterns, as indicated by the student at enrollment	Enrollment summary by school, 2018	Nominal	0 = *male*; 1 = *female*
	Age	Age of the student in years as of June 30, 2018	Enrollment summary by school, 2018	Continuous	13–82
	Education level	Education level in which the student is enrolled; could be elementary or high school in science–humanities or technical–professional	Enrollment summary by school, 2018	Nominal	1 = *elementary school*; 2 = *high school (science–humanities)*; 3 = *high school (technical–professional)*
School (structure)	Schedule	Time of day that students attend school	Enrollment summary by school, 2018	Nominal	1 = *morning*; 2 = *afternoon*; 3 = *morning and afternoon*; 4 = *evening and night*
	Average no. of students	Average number of students per classroom	Enrollment summary by school, 2018	Continuous	0–44
	No. of teachers	Total number of teachers at the school during the selected year	National performance evaluation system, 2018–2019	Continuous	2–120
	Student–teacher ratio	Number of students divided by the number of teachers	National performance evaluation system, 2018–2019	Continuous	4–27
School (management)	Additional quality-based grants	Amount of additional financial aid provided due to low performance	School grants, 2018	Ordinal	0 = *no additional grant*; 60 = *additional 60% grant*; 100 = *additional 100% grant*
	Effectiveness	The educational outcome achieved by the school in relation to its student sample	National performance evaluation system, 2018–2019	Continuous school self-reporting through a standardized evaluation card	0–100
	Achievement	The school’s educational achievement differentials over time	National performance evaluation system, 2018–2019	Continuous school self-reporting through a standardized evaluation card	0–100
	Innovation	The school’s capacity to innovate in education and engage the support of external partners for its pedagogical work	National performance evaluation system, 2018–2019	Continuous school self-reporting through a standardized evaluation card	0–100
	Improvement	The improvement of working conditions and the proper running of the school	National performance evaluation system, 2018–2019	Continuous school self-reporting through a standardized evaluation card	0–100
	Integration	Integration and participation of teachers, parents, and guardians in realizing the school’s educational goals	National performance evaluation system, 2018–2019	Continuous school self-reporting through a standardized evaluation card	0–100
	Equality	Equality in terms of the student sample being able to access and remain in the school and integration of groups with learning disabilities	National performance evaluation system, 2018–2019	Continuous school self-reporting through a standardized evaluation card	0–100

### 2.3. Procedure

After retrieving these datasets, we identified relevant variables from the codebook provided. As a starting point for the merging of databases, we used the performance summary dataset. This was merged with the enrollment summary by the school dataset through an encrypted code assigned to each student. This dataset has information on individual characteristics and the school in which each student was enrolled in 2018. To clean the resultant database, we dropped observations that had missing information on individual characteristics or where a student appeared more than once (e.g., because the student had transferred schools). The resultant sample featured 10,356 students. Then, we added students’ school information by merging the national performance evaluation system and school grants datasets with the previous merged dataset using school identification numbers. We also recoded certain variables, grouping education levels and age by school, to simplify our analyses. During this process, we cleaned our data of invalid cases with missing school information. The final sample consisted of 10,130 students.

During this process, we cleansed our data of invalid cases where student information on grades and attendance was missing or where information appeared more than once (e.g., because the student had transferred schools). In all, we discarded 62% of our original sample (*n* = 16,298).

Table 2 compares the means of the study variables between the original and final samples. The statistical significance of the mean differences was estimated through Student’s *t*-test. There were statistically significant differences between samples in all variables but GPA, the percentage of students in high school (technical–professional), percentage of students in municipal and partially subsidized schools, percentage of students with a 60% grant, and the achievement score of the students’ schools. The final sample was composed of students with a lower dropout rate, a lower proportion of men students, and lower average age. This loss of sample data due to the process of merging datasets increases bias, and therefore findings should be regarded as only representative of this sample.

**TABLE 2:** Differences between initial sample and final sample.

	Original sample (*n* = 16,298)		Final sample (*n* = 10,130)		Difference
**Variables**	**Mean (or %)**	**SD**	**Mean (or %)**	**SD**	
Dropout	31.2	0.46	6.4	0.24	24.8***
Gender (Man = 1)	58.7	0.49	50.8	0.49	7.9***
Age	25.7	10.7	24.3	9.6	1.4***
GPA	5.5	0.5	5.5	0.5	0.0
**Education level**					
Elementary school	18.0	0.38	0.0	0.02	18.0***
High school (humanities–science)	71.6	0.45	90.0	0.30	-18.4***
High school (technical–professional)	10.5	30.6	10.0	0.30	0.5
**Schedule**					
Mornings	11.8	0.32	13.0	0.33	-1.2***
Afternoons	9.8	0.30	8.0	0.28	1.8***
Mornings and afternoons	8.4	0.28	3.0	0.17	5.4***
Evenings and nights	70.0	0.46	76.0	0.43	-6.0***
**Administrative dependence**					
Municipal	89.1	0.31	89.0	0.31	0.1
Partially subsidized	10.4	0.31	10.0	0.30	0.4
Local public education service	0.5	0.07	1.0	0.08	-0.5*
Rural school (Yes = 1)	9.8	0.29	6.0	0.24	3.8***

*p < 0.05, ***p < 0.001. The *t*-tests were carried out to estimate the difference between the original and final sample.

### 2.4. Analysis plan

We performed our analyses using Stata 15 statistical software. As part of our descriptive analysis, we carried out a frequency analysis to further characterize this educational sample at the individual and school levels. We also performed a *t*-test to draw a mean comparison between students who did not complete the 2018 school year (dropped out) and those who did (passed or failed). Then, we conducted a multilevel logistic analysis, constructing four models based on the two levels. The dependent variable for this analysis was the dichotomous dropout variable (1 = dropped out, 0 = completed). Model 0 was the intercept-only model. Model 1 included the individual characteristics (level 1). Model 2 included individual variables and school-level variables (level 2), accounting for schools’ structural attributes. Finally, Model 3 included all variables from the first two models and the school-level variables characterizing school management.

### 2.5. Ethical considerations

For this study, we requested information through the mechanism provided by the Chilean Government’s Transparency Law 20.285 (2015), ensuring that it was only used for scientific purposes and safeguarding the integrity and anonymity of the schools, their students, and their performance indicators. We later consulted data provided by the Ministry of Education through its online study center, an open data website with detailed information on Chile’s education system with free access to various datasets. In both the analyses and presentation of results, attention was given to maintaining and safeguarding the privacy of these data. It should be noted that the researchers did not have access to data that could have allowed them to identify individual students because the datasets are encrypted. Although it was possible to merge the data using student identifier codes, this is an encryption of personal data and is therefore not in the public domain.

## 3. Results

Table 3 displays the descriptive results of the variables we studied, showing means for the continuous variables and percentages for the dichotomous variables. Regarding the individual variables, we found several significant differences between students who left school and those who did not, based on their own attributes. In terms of gender, more women (*p* < 0.01) dropped out than men. Students who dropped out also had a lower mean age (*p* < 0.001) and grade point average (*p* < 0.001) than those who remained in school. Finally, looking at education level, those who had a technical–professional high school education were more likely to drop out (*p* < 0.001). The mean comparison tests showed that these differences were indeed significant. Hence, to characterize our sample, students who dropped out of the education system were more likely to be women, young people, and people with low academic achievement. This occurred at a significantly higher rate among students who attended technical–professional high schools.

**TABLE 3:** Descriptive data and differences according to dropout rate and *t*-test.

	Not drop out (*n* = 9,482)		Dropped out (*n* = 648)		Total		
	***M***	***SD***	***M***	***SD***	***M***	***SD***	**Difference**
**Individual**							
Gender	0.49	0.50	0.44	0.50	0.51	0.49	0.06**
Age	24.4	9.8	22.6	7.1	24.3	9.6	1.7***
GPA	5.5	0.5	5.2	0.4	5.5	0.5	0.3***
**Education level**							
Elementary school	0.00	0.01	0.00	0.04	0.00	0.02	0.00
High school (humanities–science)	0.91	0.29	0.85	0.36	0.90	0.30	0.06***
High school (technical–professional)	0.90	0.29	0.15	0.36	0.10	0.30	-0.06***
**School (structure)**							
**Schedule**							
Mornings	0.13	0.34	0.08	0.27	0.13	0.33	0.05***
Afternoons	0.08	0.28	0.09	0.28	0.08	0.28	-0.01
Mornings and afternoons	0.03	0.17	0.04	0.19	0.03	0.17	-0.01
Evenings and nights	0.76	0.43	0.79	0.41	0.76	0.43	-0.03*
**Administrative dependence**							
Municipal	0.89	0.31	0.90	0.30	0.89	0.31	-0.01
Partially subsidized	0.10	0.30	0.09	0.29	0.10	0.30	0.01
Local public education service	0.01	0.08	0.00	0.07	0.01	0.08	0.01
**Location**							
Rural	0.06	0.24	0.05	0.22	0.06	0.24	0.01
Urban	0.94	0.24	0.95	0.22	0.94	0.24	-0.01
Average no. of students	23.5	6.7	23.9	7.3	23.5	6.7	-0.4
No. of teachers	46.7	26.7	50.6	30.6	46.9	27.0	-3.9***
Student–teacher ratio	12.3	4.0	12.2	4.4	12.3	4	0.4
**School (management)**							
**Additional grants**							
100% grant	1.9	0.3	2.0	0.2	1.9	0.3	0.0*
60% grant	2.0	0.1	2.0	0.0	2.0	0.1	0.0*
**Performance**							
Effectiveness	42.4	6.3	41.7	6.9	42.3	6.4	0.6*
Achievement	62.3	3.9	61.9	3.7	62.3	3.9	0.4*
Innovation	55.0	43.0	55.8	42.8	55.1	42.9	-0.8
Improvement	95.0	10.3	94.0	12.1	94.9	10.4	1.0*
Integration	45.0	37.4	45.4	36.9	45.0	37.3	-0.4
Equality	87.1	9.7	87.7	9.8	87.1	9.8	-0.6

The right-most column shows the difference between the means of those who did not drop out and those who did; t-tests were carried out to show if differences between those who dropped out and those who did not were statistically significant. *p < 0.05, **p < 0.01, ***p < 0.001.

Regarding the schools’ structural attributes, a Student’s *t*-test showed that students who attended school in the evening and at night dropped out more frequently (*p* < 0.05). Likewise, the results indicate that a significantly smaller proportion of students attending school in the morning dropped out a second time (*p* < 0.001).

There were no significant differences for the students’ enrollment in neither type of administrative dependence nor school location. There was a difference, however, with respect to the number of teachers per school, whereby the average was higher among students who left school again. In other words, students were more likely to drop out of schools that had more teachers (*p* < 0.001).

In terms of school management, it is worth noting that very few YAE institutions received additional quality-based grants, at neither 60% nor 100%. A Student’s *t*-test revealed significant mean differences in this respect (*p* < 0.05), indicating that people who dropped out tended to be enrolled in schools that did not receive additional funding. Regarding performance data reported by the National Performance Evaluation Service, which were based on evidence-based self-reports submitted by school principals, the results showed significant differences in school dropout based on effectiveness, achievement, and improvement (*p* < 0.05). Indeed, the higher these values were for schools, the less likely students were to drop out.

Table 4 shows the results of the estimation of the multilevel logistic regression. The dependent variable used in the estimation was the dropout status of students, controlling for individual and school factors. According to Model 1, which included the individual variables, one age group was more likely to drop out of school again compared to students between 13 and 21 years old: those between the ages of 22 and 30 (OR = 1.27, *p* ≤ 0.05). Grade point average also appeared to affect dropout rates (OR = 0.31, *p* < 0.001); specifically, the lower the GPA, the higher the likelihood of dropping out.

**TABLE 4:** Results of multilevel logistic regression of dropout based on individual and school-level variables (*N* = 10,130 students at 179 schools).

	Model 0		Model 1		Model 2		Model 3	
	***OR***	***SD***	***OR***	***SD***	***OR***	***SD***	***OR***	***SD***
**Individual**								
Gender			1.05	0.09	1.04	0.09	1.04	0.08
**Age range (ref: 13–21 years)**								
22–30 years			1.27*	0.15	1.28*	0.14	1.29*	0.15
31–40 years			0.88	0.16	0.88	0.15	0.90	0.16
41 years or older			0.74	0.17	0.74	0.17	0.74	0.17
GPA			0.31***	0.03	0.29***	0.03	0.29***	0.03
**School**								
Average no. of students					1.01	0.02	1.00	0.01
No. of teachers					1.00	0.00	1.00	0.00
Enrollment					0.99	0.00	0.99	0.00
Student–teacher ratio							20.6***	19.2
**Administrative dependence (ref: municipal)**								
Partially subsidized					0.68	0.20	0.68	0.20
Local public education service					1.62	0.75	1.17	0.59
**Education level (ref: elementary school)**								
High school (humanities–science)					0.10*	0.12	0.10*	0.11
High school (technical–professional)					0.24	0.29	0.20	0.25
Rural school (1 = *yes*)					1.08	0.30	1.00	0.28
**Schedule (ref: morning)**								
Afternoon					1.74**	0.39	1.75**	0.39
Morning and afternoon					1.08	0.44	1.23	0.51
Evening and night					1.52*	0.26	1.49*	0.29
**Additional quality-based grants**								
No 100% grant					1.63**	0.32	1.31	0.38
No 60% grant					12.02***	8.68	10.67***	7.94
**Performance**								
Effectiveness							1.00	0.01
Achievement							0.96**	0.01
Innovation							1.01	0.00
Improvement							1.01	0.00
Integration							0.98*	0.00
Equality							1.01	0.00
Constant	0.06***	(0.00)	30.82***	16.43	8.94	13.30	17.75	38.00
**Variance partitioning coefficient**								
School level	0.111		0.108		0.086		0.079	
Log-likelihood	−2,360.58		−2,269.01		−2,248.11		−2,243.22	

*p < 0.05, **p < 0.01, ***p < 0.001.

For Model 1, the null hypothesis was rejected for age and GPA because students between 22 and 30 years of age and those with low grades were more likely to drop out of school again. The null hypothesis was accepted for gender, however, because there were no significant differences in this respect in the sample analyzed. In this line, the data may not have had enough power to detect this association.

Model 2 included the individual variables and the variables relating to school structure. It should be noted that adding these variables did not change the significance of the individual variables noted in the previous model. Thus, Model 2 showed no significant differences in terms of student enrollment, average number of students per classroom, or number of teachers. However, a difference emerged for the student–teacher ratio. Indeed, the higher the ratio, the more likely students were to drop out of school again (OR = 19.96, *p* < 0.001).

Regarding students’ school schedule, those who attended in the afternoon (OR = 1.78, *p* ≤ 0.01) and those who attended in the evening and at night (OR = 1.52, *p* ≤ 0.05) were significantly more likely to drop out of school than those who attended in the morning. The model also showed higher dropout rates in schools without an additional 100% grant (OR = 1.63, *p* ≤ 0.01) and those without an additional 60% grant (OR = 12.02, *p* < 0.001). In other words, schools that lacked these additional resources were more conducive to students dropping out.

Finally, we added the variables relating to institutional management to produce Model 3. From this model, we ascertained whether school performance explained dropout, per the National Performance Evaluation System criterion. It should be noted that when the management variables were added, the odds’ ratio for whether schools had a 100% grant was no longer significant. As for the management variables, achievement (OR = 0.96, *p* ≤ 0.01) and integration (OR = 0.99, *p* ≤ 0.05) predicted dropout. In this regard, students were less likely to drop out of schools with higher educational achievement differentials over time and those with higher levels of teacher integration and participation.

In summary, school variables such as when students attended class, the grants that schools received, educational achievement, and teacher integration were shown to affect the likelihood of dropout. The null hypothesis was rejected because no significant differences were found for variables such as the average number of students, number of teachers, and other management-related factors.

In order to calculate the variance partition coefficient (VPC), we used Snijders and Bosker (2012) approach known as the latent variable method. The VPC allows quantifying the proportion of the total variance that is attributable to the different levels in the model for a given set of variables (Goldstein et al., 2002).

The VPC for Model 0 (intercept-only model) showed that 88.89% of the variance in students dropping out of YAE was explained by individual variables, whereas 11.11% of the variance was explained by school-related attributes. In Model 3, the VPC for level 2 (school-level) dropped to 7.9%, showing the relevance of individual characteristics. Therefore, we found support for the hypothesis that variance in school dropout would be more greatly explained by individual factors than by differences between YAE institutions.

## 4. Discussion

Returning to school after having left regular education by entering a YAE school does not guarantee graduating and, thus, finishing the compulsory school trajectory. In fact, dropping out of a YAE school constitutes, for these students, a second school dropout experience that ultimately hinders their “last chance” possibility of graduating from high school. In this vein, this study sought to analyze individual and school-level factors that, together, contribute to explaining why students who have already dropped out (at least once) of school and are willing to try again in a YAE school may drop out of YAE too, increasing the risk of not completing their formal education.

Our findings show that in terms of students’ individual attributes, there are gender differences in school dropout. Although the literature has reported that men drop out the most, our analysis suggested that women were more likely to drop out of school again in the context of YAE. Although this variable was no longer significant in Model 2, our results are nevertheless consistent with previous research showing that young women from vulnerable communities are sometimes more likely to have their educational journeys cut short (Hubert et al., 2019). This can be largely explained by teenage pregnancy, but research has also identified financial instability, various types of violence, and lack of backing from institutions as additional factors (Vázquez-Nava et al., 2019). Support for these women should be sought from a gender perspective, with a focus on keeping them in school and, in so doing, closing the significant gap in this respect (Banda et al., 2019).

Our results also indicate that the highest dropout rates and poorest school performance in YAE occurred among young people. These data suggest that significant efforts are required to meet the needs of these young people, who continue to be outsiders in school and, thus, have dropped out a second time. To delve further into this matter, syllabus design must be analyzed. In addition, teaching methodologies and assessment models should be examined to identify factors that need to be addressed to support these students and promote learning and participation (Allison and Attisha, 2019; Låg and Sæle, 2019).

At the school level, the management, and accountability indicators of achievement and integration could have a significant impact on school dropout. As these indicators increased, the likelihood of YAE students dropping out of school again decreased. The achievement indicator reflects the schools’ educational achievements over time and, thus, can be taken as a proxy for academic performance. Students are more likely to complete their compulsory education in high-performing schools. Contrarily, students have a tougher time continuing their education in schools that do not do so well in this respect, thus perpetuating school exclusion and negative experiences (Warne et al., 2020).

Our findings regarding school integration are also noteworthy. This indicator reflects teacher participation and opportunities for parents, guardians, and students to lead and participate. As such, it considers multiple stakeholders in the school community. This management indicator also measures educational commitment, based on the type and number of activities organized (Lee and Desjardins, 2019). Therefore, it is a fairly broad indicator, accounting for multiple practices in the school environment that may diminish the likelihood of students dropping out. Considering our findings, we can identify strategies that address dropout from a relational perspective in the school environment (Itzhaki, 2019). These strategies should take a systemic, inclusive approach that emphasizes the construction of democratic and participatory spaces (De la Cruz Flores and Ortega, 2019; Portela et al., 2019).

In terms of school management, it is evident that more resources are needed to support learning processes because this could reduce the number of students who discontinue their schooling. These resources should include physical improvements to school facilities and more support for students in terms of food, transportation, and educational materials, thus encouraging and helping them remain in the education system. However, an enormous risk for YAE school dropout is presented by the effects of distance learning education that has been carried out in most YAE schools in Chile during the COVID-19 pandemic. Unlike regular K-12 schools, which have progressively advanced toward in-person learning with safety measures, YAE schools have been physically closed for almost 2 years, with students receiving guides and materials that they must complete alone at home. This likely has had a large effect on the relational perspective (Itzhaki, 2019) that young, lower-achieving students in YAE schools need to engage and keep studying in these schools.

At the school level, the teacher–student ratio was found to be a determining factor in school dropout. The literature has already noted that personalized work helps keep students in school, given the social support that they perceive from their teachers in such cases. Importantly, the climate and relationships that this approach creates translate into greater school commitment (Terrenghi et al., 2019) and transform the educational experience, promoting students’ continued engagement and keeping them from dropping out.

Following the latent variable method used by Snijders and Bosker (2012), the variance partition coefficient (VPC) was estimated in order to quantify the total variation attributable to differences between schools. The VPC in the estimated models had values in the range of [0.079–0.108]—in other words, between 7.9 and 11.1% of the total variation could be attributable to level 2 differences, showing that a high proportion of the unexplained variance is related to the differences between students’ characteristics within schools.

Because individual factors made the highest contribution to explaining school dropout in this national sample of students, there is a risk of putting all the responsibility on students. However, as stated previously, the analysis and possible lines of action and intervention cannot be reduced to one level, given the complex nature of school dropout. Research on school improvement has shown that schools can and do make a difference when they provide opportunities for inclusion, learning, and participation for all students (Ainscow, 2015). The findings from this study suggest that school interventions and public policies based on school improvement and educational inclusion may strengthen the YAE school experience such that student characteristics may have lower predictive power, and in contrast, school characteristics, particularly school management practices, may play a greater role in preventing school dropout. To foster this transformation, researchers and educational communities should focus on both the strengthening and visibility of YAE, as well as on facilitating evidence-based decisions that consider the situated indicators that predict YAE student dropout (Rodríguez et al., 2023).

Summing up, school dropout leads to exclusion and has direct repercussions on the educational attainment, life projects, and subjectivities of the students affected. Although attention is often placed on the mainstream education system, school dropout also occurs in educational settings for students that have already left school at least once.

Returning to the questions that guided this study—namely, what variables are decisive in understanding early school leaving in YAE and what actions can be deployed to intervene in this phenomenon—we can conclude that school dropout in YAE can only be adequately explained by considering individual, school, and social factors jointly (Prenkaj et al., 2020). Our results reveal individual risk factors such as age and low grades and protective factors that help to build connections and foster student engagement, such as the teacher–student ratio at the school, available resources, and how the school environment is managed. By addressing these issues, recognizing the needs of students, and giving them the right support, we can change their lives. Not only will they receive their right to education, but they will also gain new opportunities to shape their future and realize their life goals.

Even so, many obstacles remain that push these young adult and adult students to drop out of school again. To reverse this situation, we propose searching for other non-privatizing models, which have dominated Chile’s education system for over forty years as a result of neoliberal logic, affecting the most vulnerable students, such as those in YAE. These educational models should focus on gender differences, context-specific curricular and assessment plans, and school management that promotes inclusivity, democratic school experiences, and student engagement. Considering these variables from a wellbeing and social justice framework, the consequences and effects of school dropout on young and adult people could be modified, configuring new educational trajectories.

Among the limitations of this study, although the sample was national, the dataset analyzed represented a subsection of the total universe of students enrolled in YAE schools in Chile. The final sample analyzed also differed in the proportion of men students, ages, and grades at the individual level, and the students’ schools differed in structural characteristics from the original sample. The datasets analyzed did not include information regarding student engagement, participation, or motivation toward learning. Another important limitation to address in follow-up studies is the lack of monitoring and updating of the YAE datasets since the COVID-19 pandemic. It is due to this that it has not been possible to carry out a cross-over study. Therefore, we suggest that future studies include a cross-sectional study over time and broaden the scope of analysis, data, and information of the experience of leaving school, such as the educational and socioemotional effect, for comprehending the factors involved in YAE school dropout. Finally, although this study contributes to the existing scientific evidence by incorporating contextual, school-related attributes that contribute to explaining school dropout in the YAE educational setting, future studies should complement these analyses by incorporating other algorithmic and machine learning approaches.

## Data availability statement

The original contributions presented in this study are included in the article/supplementary material, further inquiries can be directed to the corresponding author.

## Ethics statement

The studies involving human participants were reviewed and approved by the Pontificia Universidad Católica de Valparaíso. Written informed consent for participation was not required for this study in accordance with the national legislation and the institutional requirements.

## Author contributions

TC-V: conceptualization, formal analysis, methodology, writing—original draft, and writing—review and editing. VL: conceptualization, writing—review and editing, supervision, and project administration. EB: methodology, writing—original draft, and writing—review and editing. LG: methodology and formal analysis. All authors contributed to the manuscript revision, read, and approved the submitted version.
